# Comparative Study on the In Vitro Gastrointestinal Digestion of Oil Body Suspension from Different Parts of *Idesia polycarpa* Maxim

**DOI:** 10.3390/gels12010073

**Published:** 2026-01-14

**Authors:** Silu Cheng, Yongchen Liu, Mingzhang Zhao, Shanshan Qian, Hongxia Feng, Yunhe Chang, Juncai Hou, Cong Xu

**Affiliations:** 1 College of Food Science and Engineering, Guiyang University, Guiyang 550005, China; 2 Key Laboratory of Dairy Science, College of Food Science, Northeast Agricultural University, Harbin 150030, China

**Keywords:** *Idesia polycarpa* Maxim, oil body, in vitro digestion, antioxidant capacity

## Abstract

This study provides the first comparative analysis of the physicochemical and functional properties of oil body suspensions derived from different parts—entire fruit (EOB), peel (POB), and seed (SOB)—of *Idesia polycarpa* Maxim (IPM) during in vitro simulated gastrointestinal digestion. Results demonstrated that the properties of the different suspensions exhibited significant difference during digestion stages. The average particle size of all suspensions decreased, with the most significant reduction observed in POB (91.50%), which was attributable to its lower interfacial protein content and inferior stability. The absolute ζ-potential decreased in the model of gastric digestion (MGD) due to interface disruption but increased in the model of intestinal digestion (MID) following the adsorption of bile salts. Throughout the simulated digestion process, the protein hydrolysis degree, free fatty acid (FFA) release rate, reducing power, and inhibition rates against α-amylase and α-glucosidase all increased, concurrently with a decrease in DPPH radical scavenging activity. Notably, the POB suspension exhibited the highest extent of lipid digestion, with the highest cumulative FFA release rate (27.83%). In contrast, the SOB suspension showed the most significant enhancement in total reducing power (increased by 199.32% after intestinal digestion) and the highest α-glucosidase inhibitory activity. These findings clarify that the part source is a critical factor influencing the digestive properties and functional activities of IPM oil bodies, providing a theoretical foundation for the targeted application in functional foods.

## 1. Introduction

*Idesia polycarpa* Maxim (IPM) is a promising woody oil species, valued for its high yield, strong adaptability, and high oil content [1]. Compared to woody oil plants such as olive, oil camellia, and oil palm, IPM is exceptionally rich in linoleic acid, with a content of approximately 63.58%, which is comparable to that of sunflower seed oil [2,3]. Meanwhile, IPM is rich in nutrients such as vitamin E, squalene, and polyphenols, contributing to its high nutritional value [4]. In addition, IPM also exhibits excellent antioxidant activity and can improve lipid metabolism, offering potential in the prevention and treatment of non-alcoholic fatty liver disease [5]. Consequently, IPM has attracted growing interest for its potential in functional food applications.

Fat-soluble nutrients, which encompass fat-soluble vitamins, polyunsaturated fatty acids, carotenoids, and curcumin, play important roles in human health. Studies have shown that fat-soluble nutrients can prevent and control various chronic diseases, such as cardiovascular disease [6], diabetes [7,8], and cancer [9]. Fat-soluble molecules are insoluble in blood, they must be incorporated into lipoprotein particles for absorption and systemic transport [10]. Oil bodies are subcellular organelles for oil storage in plants, with a structure consisting of a triacylglycerol core surrounded by a monolayer of phospholipids, and they can serve as carriers for functional nutrients [11,12]. In our previous studies, we observed compositional and structural differences among oil bodies extracted from different parts of IPM [13]. Compared to entire fruits oil bodies (EOB) and peel oil bodies (POB), seed oil bodies (SOB) exhibited significantly higher protein content, interfacial protein adsorption capacity and a notably smaller particle size. Meanwhile, POB were characterized by a lower content of total sugars and total phenolics, but a higher lipid content than that of the EOB and SOB. These differences affect the oil bodies’ functionality as a carrier. Zhang et al. [14] found that the oil bodies with the highest surface membrane protein content and the smallest particle size exhibited a high encapsulation efficiency (91.87%) and bioavailability (91.3%) for curcumin. Investigating the digestive behavior of oil bodies is crucial for guiding their development in food and nutritional applications. Yang et al. [15] demonstrated that soybean oil bodies with different extrinsic protein contents and particle sizes exhibit significant differences in protein hydrolysis rates and free fatty acid release patterns during in vitro gastrointestinal digestion. Similarly, Liu et al. demonstrated that difference in protein composition and interfacial properties can affect the release rate of free fatty acids in oil bodies, thereby impacting their performance as delivery carriers [16].

In addition, studies have shown that oleogels prepared from oil bodies can be used as medicine carriers. Farooq et al.’s study demonstrated that oleogels constructed based on camellia oil bodies can efficiently encapsulate curcumin with an encapsulation efficiency of up to 92.18% and exhibit excellent structural stability and sustained release characteristics under simulated gastrointestinal conditions [17]. Rahman et al.’s study confirmed that oleogels based on peanut oil bodies exhibited a curcumin loading capacity of 0.9 mg/g and an encapsulation efficiency as high as 83.2%, capable of targeted delivery of curcumin to the intestines and serving as a highly promising drug delivery carrier [18]. Therefore, investigating the digestive properties of oil body suspensions is crucial for optimizing the formulation of oleogels, as it provides essential insights into how to enhance the bioavailability of encapsulated bioactives and how to tailor the targeted delivery performance of oleogel-based carriers.

The digestion behavior and properties of oil body suspensions derived from IPM have not been previously investigated. Therefore, we investigated the physicochemical and functional properties of EOB, FOB, and SOB suspensions following simulated gastrointestinal digestion, providing theoretical support for the functional application of IPM oil bodies.

## 2. Results and Discussion

### 2.1. Particle Size and ζ-Potential

**Figure 1:**
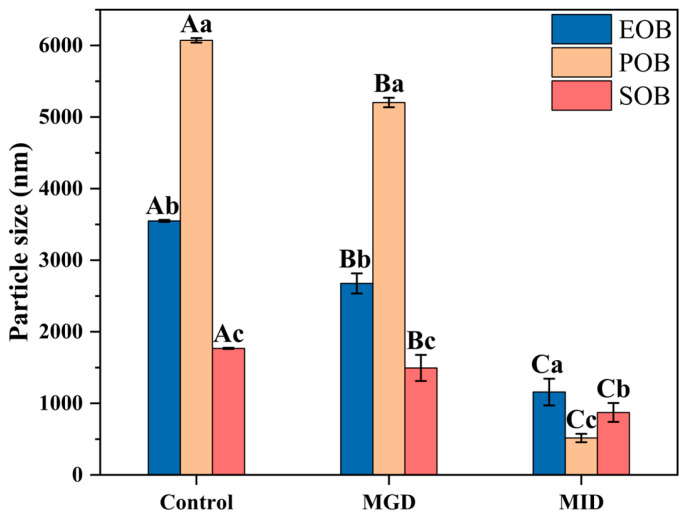
Effects of in vitro gastrointestinal digestion on particle size of IPM oil body suspension. Different uppercase letters (A, B, and C) represent significant differences (*p* < 0.05) in particle size within digestion for the same sample. Different lowercase letters (a, b, and c) indicate significant differences (*p* < 0.05) in particle size among different samples at the same digestion stage. MGD and MID are abbreviations for model of gastric digestion and model of intestinal digestion.

Particle size and ζ-potential were critical indicators for evaluating suspension stability, interfacial composition changes, and digestive characteristics. Figure 1 shows the changes in the particle size of the EOB, POB, and SOB suspensions at different stages of in vitro digestion. Before in vitro digestion, the particle size of the IPM oil body suspensions followed the order: POB (6072.00 ± 31.56 nm) > EOB (3548.67 ± 13.84 nm) > SOB (1766.67 ± 8.41 nm). After simulated gastrointestinal digestion, the particle sizes of EOB, POB, and SOB decreased by 67.37%, 91.50%, and 50.66%, respectively. The significant reduction in particle size (*p* < 0.05) after simulated intestinal digestion was attributed to the elevated pH-induced electrostatic repulsion, combined with the disruption of the oil body structure by bile salts and pancreatic lipase [19]. Meanwhile, the POB suspension exhibited the most significant reduction particle size. This is presumably attributed to its lower protein content compared to the EOB and SOB suspensions, resulting in poorer interfacial stability and heightened susceptibility to disruption by enzymes and bile salts during digestion [20,21]. Li et al.’s study [22] also demonstrated that a low protein content significantly reduced interfacial stability by impairing the mechanical strength and adsorption capacity of the interfacial film.

**Figure 2:**
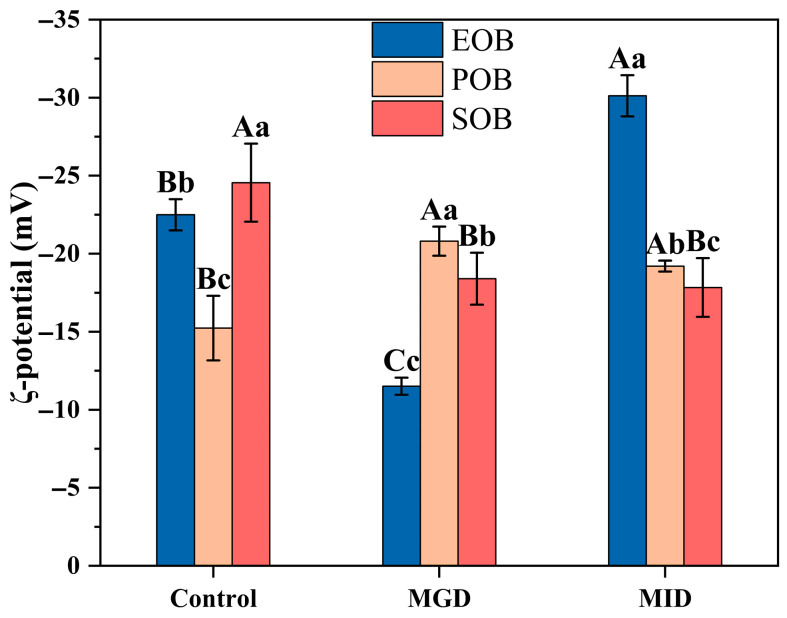
Effects of in vitro gastrointestinal digestion on ζ-potential of IPM oil body suspension. Different uppercase letters (A, B, and C) represent significant differences (*p* < 0.05) in ζ-potential within digestion for the same sample. Different lowercase letters (a, b, and c) indicate significant differences (*p* < 0.05) in ζ-potential among different samples at the same digestion stage.

Figure 2 shows the ζ-potential of the EOB, POB, and SOB suspensions during simulated digestion. In the control group, the absolute value of ζ-potential of the SOB suspension was the highest, while that of the POB suspension was significantly lower than that of the other groups (*p* < 0.05). The absolute values of ζ-potential of all oil body suspensions decreased during MGD and increased during MID. The decrease in the ζ-potential during MGD was attributed to the synergistic effect of low pH and pepsin, which disrupted the interfacial structure of the oil bodies and attenuated electrostatic repulsion, thereby reducing suspension stability [23]. During MID, the adsorption of bile salts and digestion-induced reduction in suspension particle size (Figure 1) contributed to the enhanced stability of the system.

### 2.2. In Vitro Protein Hydrolysis Degree and Free Amino Acid Analysis

**Figure 3:**
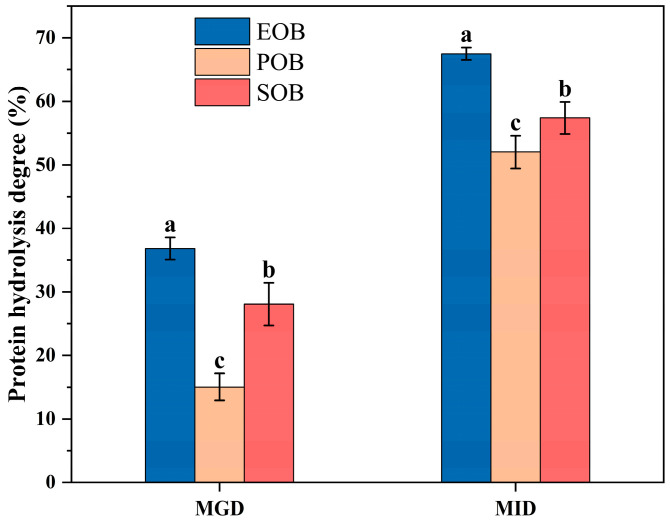
Effects of in vitro gastrointestinal digestion on protein hydrolysis degree of IPM oil body suspension. Different lowercase letters (a, b, and c) indicate significant differences (*p* < 0.05) in protein hydrolysis degree among different samples at the same digestion stage.

Interfacial proteins are important for maintaining the structural stability of oil bodies. During digestion, a higher degree of hydrolysis of these proteins indicates more disruption of the oil body structure. This facilitates the release of internal lipids, exposing them to digestive enzymes and potentially enhancing the bioavailability of fat-soluble nutrients. The protein hydrolysis degree was calculated using the initial protein content of each emulsion as the baseline, eliminating the interference of initial content variations on the comparison of hydrolysis efficiency. The initial protein content of three samples were as follows: SOB (12.00 ± 0.37%) > EOB (7.46 ± 0.50%) > POB (5.71 ± 0.23%). The protein hydrolysis degree of the EOB, POB, and SOB suspensions are shown in Figure 3. In MGD, the hydrolysis degree of the oil body suspensions ranged from 15.02 ± 2.13% to 36.82 ± 1.76%, with EOB exhibiting a significantly higher hydrolysis degree (36.82 ± 1.76%) than the other groups (*p* < 0.05). In MID, the hydrolysis degree of the oil body suspensions of all groups increased, ranging from 52.03 ± 2.57% to 67.50 ± 0.99%, and EOB suspension maintaining the highest hydrolysis degree. These results indicate that the interfacial proteins of IPM oil bodies from different parts exhibited varying susceptibility to gastrointestinal enzymes. Furthermore, intestinal digestion exerted a stronger promoting effect on the hydrolysis of the interfacial proteins of IPM oil bodies suspensions than gastric digestion. In Lin et al.’s study [24], it was also demonstrated that intestinal digestion can hydrolyze interfacial proteins into small peptides and amino acids more efficiently, with a much higher degree of hydrolysis than that of gastric digestion.

**Figure 4:**
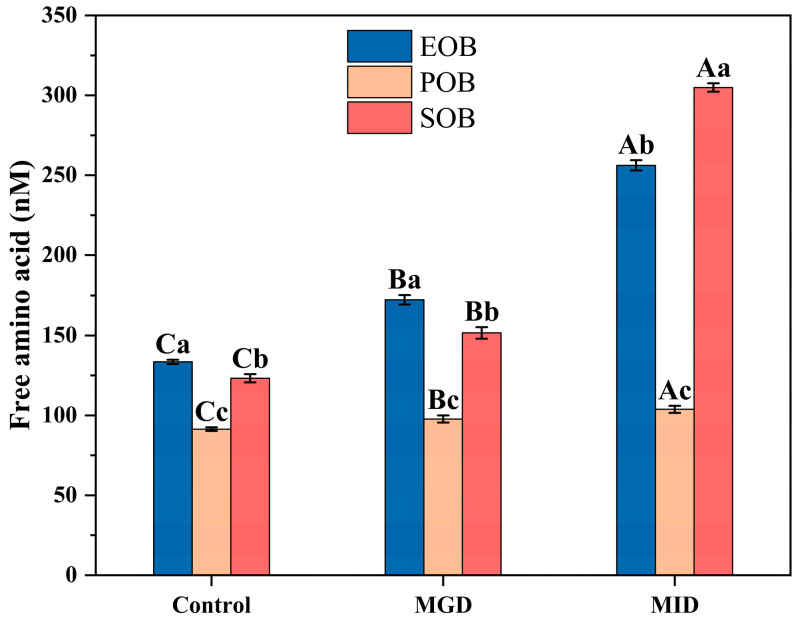
Effects of in vitro gastrointestinal digestion on free amino groups of IPM oil body suspension. Different uppercase letters (A, B, and C) represent significant differences (*p* < 0.05) in free amino groups within digestion for the same sample. Different lowercase letters (a, b, and c) indicate significant differences (*p* < 0.05) in free amino groups among different samples at the same digestion stage.

Free amino groups were produced during the protein hydrolysis of oil body suspensions, and their concentration was used to indicate the protein hydrolysis degree of the suspensions (Figure 4). During gastrointestinal digestion, the free amino group contents of EOB, POB, and SOB increased by 92.48%, 13.18%, and 147.15%, respectively. The increase in free amino group content during intestinal digestion was much greater than that during gastric digestion, which was consistent with the protein hydrolysis degree results. This phenomenon aligns with the findings of Fan et al. [25], who demonstrated that the intestinal digestion condition exhibits more vigorous digestive activity than the gastric condition. Regarding the apparent contradiction that EOB exhibited higher overall protein hydrolysis degree but a lower increase in free amino groups compared to SOB, this phenomenon is attributed to differences in the composition and molecular weight of the proteins in the two oil bodies.

### 2.3. Free Fatty Acid Release Rate

**Figure 5:**
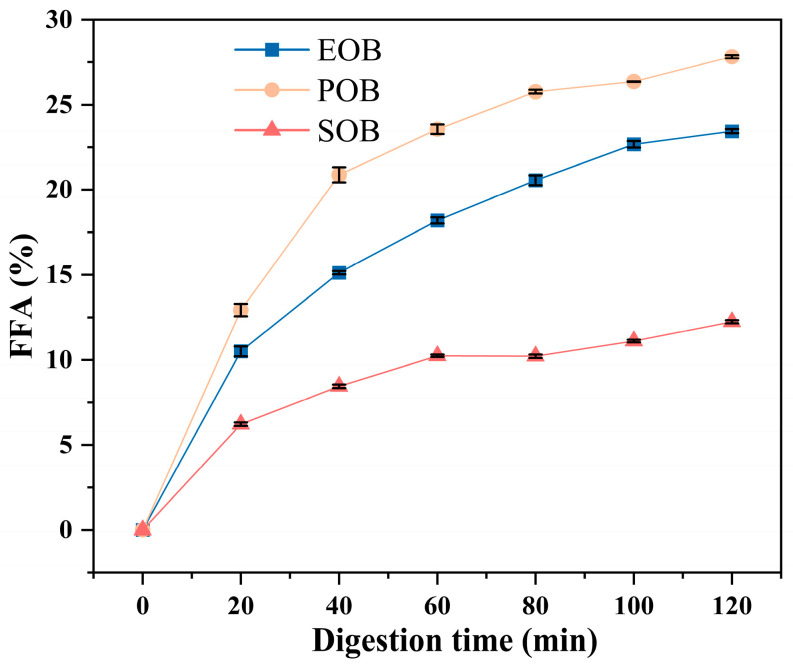
Free fatty acid release rate of IPM oil body suspension during in vitro gastrointestinal digestion.

The free fatty acid (FFA) release rate is a key indicator for assessing the hydrolysis efficiency of triglycerides in oil bodies by pancreatic lipase, serving as a crucial parameter for evaluating lipid nutrient absorption. Figure 5 shows the FFA release rate from IPM oil body suspensions during a 120 min in vitro digestion. All suspensions exhibited a rapid initial increase in FFA release from 0 to 40 min, which was followed by a markedly slower release phase from 60 to 120 min. The POB suspension demonstrated the highest cumulative FFA release (12.93 ± 0.36–27.83 ± 0.09%), which consistently exceeded that of the EOB (10.50 ± 0.29–23.44 ± 0.11%) and SOB (6.22 ± 0.10–12.24 ± 0.09%) suspensions during the entire process. With the extension of digestion time, triacylglycerols (TAGs) in the oil bodies were continuously hydrolyzed by pancreatic lipase, leading to the gradual release of FFAs. The significant differences in FFA release rates among the three suspensions were mainly attributed to their inherent compositional and structural disparities. Specifically, the SOB suspension had the lowest FFA release rate, which related to its higher interfacial protein content and smaller particle size. Similarly, Yang et al. found that soybean oil bodies with higher interfacial protein content and smaller particle size exhibited a lower FFA release rate [15]. Notably, the FFA release rate of the IPM oil body suspension was significantly lower than that of common edible oils such as palm oil, rapeseed oil, soybean oil, and coconut oil [26,27]. Its unique slow-digestion property effectively mitigated postprandial blood lipid fluctuations caused by rapid fatty acid absorption, while prolonging the intestinal retention time of fat-soluble sensitive components to reduce degradation loss. This endowed it with remarkable application advantages in the development of functional foods for blood lipid control and the targeted delivery of fat-soluble nutrients.

### 2.4. DPPH Radical Scavenging Activity

**Figure 6:**
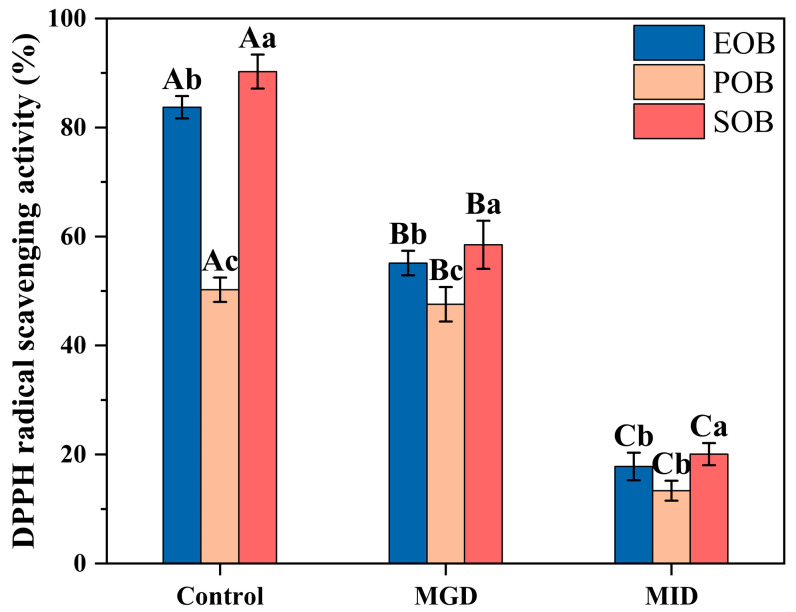
Effects of in vitro gastrointestinal digestion on DPPH radical scavenging activity of IPM oil body suspension. Different uppercase letters (A, B, and C) represent significant differences (*p* < 0.05) in DPPH radical scavenging activity within digestion for the same sample. Different lowercase letters (a, b, and c) indicate significant differences (*p* < 0.05) in DPPH radical scavenging activity among different samples at the same digestion stage.

The DPPH radical scavenging activity assesses the free radical scavenging ability of antioxidant components (such as phenolics and vitamin E) in oil bodies, serving as a reference for their application in antioxidant-functional foods. Figure 6 illustrates the DPPH radical scavenging activity of the IPM oil body suspensions during simulated gastrointestinal digestion. The DPPH radical scavenging activity of the suspensions decreased during both MGD and MID. Compared to the in vitro gastric condition, the DPPH radical scavenging activity of the EOB, POB, and SOB suspensions decreased by 37.31%, 34.20%, and 38.42%, respectively, after in vitro intestinal digestion. This reduction was attributed to the disruption of the oil body structure, which further compromised the stability of the antioxidant compounds, ultimately leading to a reduction in their activity. Phenolic compounds and proteins are natural antioxidants, and their oxidative activity is related to their molecular structures [28]. The decrease in DPPH radical scavenging activity might be attributed to the structural destruction of proteins and phenolic compounds in IPM oil bodies caused by digestion. Furthermore, the decrease in DPPH radical scavenging activity might also be attributed to the hydrolysis of proteins into short peptides and amino acids, which increased the polarity of the suspension and reduced the reaction efficiency with lipid-soluble DPPH radicals [29,30,31].

### 2.5. Reducing Power Assay

**Figure 7:**
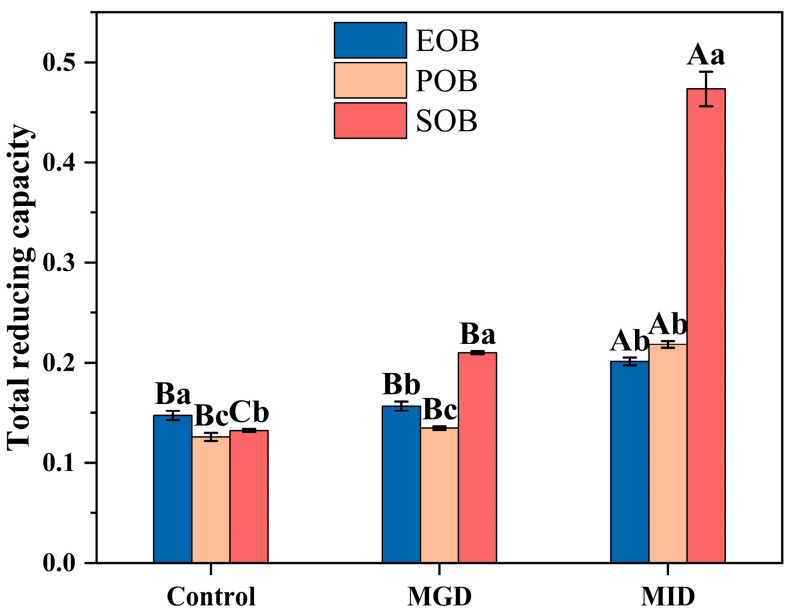
Effects of in vitro gastrointestinal digestion on total reducing power of IPM oil body suspension. Different uppercase letters (A, B, and C) represent significant differences (*p* < 0.05) in total reducing power within digestion for the same sample. Different lowercase letters (a, b, and c) indicate significant differences (*p* < 0.05) in total reducing power among different samples at the same digestion stage.

Total reducing power of IPM oil body suspensions during simulated gastrointestinal digestion was measured through dynamic changes in their electron/hydrogen-donating capacity. This parameter is closely linked to the oil bodies’ antioxidant potential, an essential trait for functional food applications. Figure 7 presents the total reducing power of the IPM oil body suspensions during simulated gastrointestinal digestion. During the MGD, no significant change was observed in the reducing power of EOB and POB suspensions. Compared to the undigested samples, the reducing power of EOB, POB, and SOB increased by 36.66%, 73.25%, and 199.32%, respectively, after simulated intestinal digestion. The reducing power mainly depends on the presence of electron/hydrogen-donating substances [32]. The increase during the simulated intestinal digestion condition might have been attributed to the disruption of the oil body structure, which released internal reductive substances such as phenolics and vitamin E that could donate electrons/hydrogen atoms. Among the three samples, SOB exhibited the most significant enhancement in reducing power, indicating that its internal reductive components are more effectively released during digestion.

### 2.6. α-Amylase and α-Glucosidase Inhibition Rate

**Figure 8:**
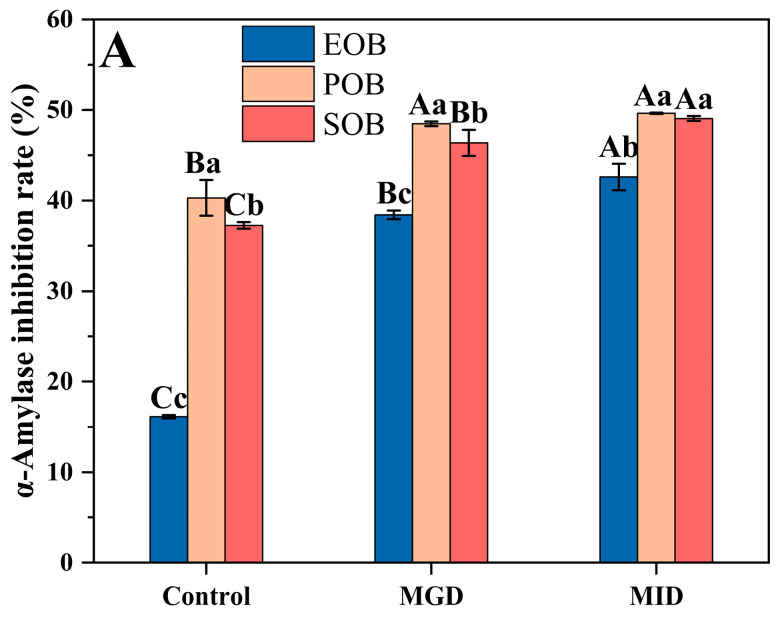

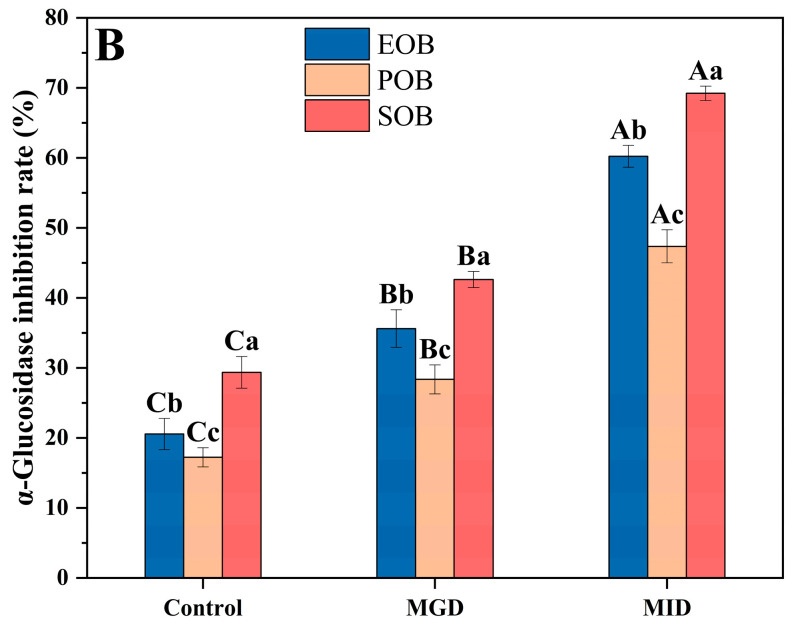
Effects of in vitro gastrointestinal digestion on α-Amylase (**A**) and α-Glucosidase (**B**) inhibition rate of IPM oil body suspension. Different uppercase letters (A, B, and C) represent significant differences (*p* < 0.05) in α-amylase inhibitory rate or α-glucosidase inhibitory rate within digestion for the same sample. Different lowercase letters (a, b, and c) indicate significant differences (*p* < 0.05) in α-amylase inhibitory rate or α-glucosidase inhibitory rate among different samples at the same digestion stage.

The α-amylase and α-glucosidase inhibition rate evaluate the inhibitory effect of oil bodies on the key enzymes involved in carbohydrate digestion, reflecting their potential blood glucose regulatory function. Figure 8 illustrates the changes in α-amylase(A) and α-glucosidase(B) inhibition rate of the suspension during simulated gastrointestinal digestion. As simulated gastrointestinal digestion proceeded, the α-amylase inhibition rate of the oil body suspension gradually increased. Compared with the control group, after the end of simulated intestinal digestion, the α-amylase inhibition rates of EOB, POB, and SOB suspensions had increased by 26%, 9%, and 18%, respectively. The α-glucosidase inhibition rate also showed a gradual increase, maintaining a consistent order throughout the process: SOB > EOB > POB. The enhancement in inhibitory activity against both α-amylase and α-glucosidase can be attributed to the disintegration of the oil body structure during digestion, which releases enzyme-inhibiting substances, including short peptides and phenolic compounds [33,34,35].

## 3. Conclusions

This study systematically investigated the in vitro digestive characteristics of oil body suspensions from IPM, obtained from the entire fruit (EOB), peel (POB), and seeds (SOB). After simulated gastrointestinal digestion, the average particle sizes of all suspensions decreased, with POB exhibiting the most significant reduction in particle size (*p* < 0.05) due to its lower interfacial protein content. During digestion, the free amino acid contents and free fatty acid release rate of the three suspensions increased. Meanwhile, POB showed the highest free fatty acid release rate. In terms of functional properties, the DPPH radical scavenging activity decreased after digestion, while their reducing power increased significantly, especially for SOB. Additionally, the α-amylase and α-glucosidase inhibition rates of the suspensions increased over digestion time, with SOB maintaining the highest α-glucosidase inhibition rate throughout the entire process. This study further advances the intensive processing of IPM. Based on their digestion profiles, POB is suitable for rapid lipid delivery, a carrier for lipophilic actives, while SOB shows potential in functional foods and sustained-release formulations due to its retained bioactivity post-digestion. It should be noted that this research is based on an in vitro model, and thus the findings require further validation in more complex physiological environments. The differences in the digestive behaviors of these oil body suspensions provide a theoretical foundation for the development of oleogels with tailored release profiles and functional characteristics. Additionally, the study did not investigate the potential effects of long-term storage on the digestive properties of the oil bodies, which will be an important direction for future work.

## 4. Materials and Methods

### 4.1. Materials

*Idesia Polycarpa* Maxim was purchased from Xinhuanfeng Agricultural Development Co., Ltd. (Zunyi, China). The PC 900 antibacterial agent was acquired from Beijing Lanjieke Technology Co., Ltd. (Beijing, China). Sucrose was purchased from Tianjin Tianli Chemical Reagent Co., Ltd. (Tianjin, China). Methanol was obtained from Tianjin Fuyu Fine Chemical Co., Ltd. (Tianjin, China). Trichloroacetic acid was purchased from Tianjin Aopusheng Chemical Co., Ltd. (Tianjin, China). Sodium dodecyl sulfate (SDS) was purchased from Saiguo Technology Co., Ltd. (Guangzhou, China). Protein Quantification Kit (BCA) purchased from Jiancheng Bioengineering Institute (Nanjing, China). Pepsin, pancreatin from porcine pancreas, *α*-amylase, and *α*-glucosidase were purchased from Sigma-Aldrich (St. Louis, MO, USA). Bile salts and DPPH reagent were purchased from Solarbio (Beijing, China).

### 4.2. Preparation of Oil Body Suspension of IPM

The preparation method of the oil bodies from IPM was based on the previous report by Qian et al. [36]. The 300 g each of the entire fruit, peel, and seeds of IPM which were artificially separated, were immersed in deionized water at a solid-to-liquid ratio of 1:6 at 4 °C for 20 h. The filtered pulp of IPM, obtained after blending for 3 min, was mixed with a 20 wt% sucrose solution. The pH of the mixture was adjusted to 7.0, followed by centrifugation (H1750R, Xiangyi Centrifuge Instrument Co., Ltd., Changsha, China) at 10,000 rpm for 30 min at 4 °C. The upper cream layer was collected and redissolved in a 20 wt% sucrose solution (pH 7.0), and the centrifugation at 10,000 rpm for 30 min at 4 °C. The washing procedure was performed three times in total, using the 20 wt% sucrose solution for the first two washes and deionized water for the final wash. The prepared 20% oil body suspension, placed in a stoppered container, was thermally treated at 90 °C for 20 min to inactivate enzymes and subsequently cooled with running water.

### 4.3. In Vitro Digestive Properties

The static in vitro digestion protocol refers to the method of Brodkorb et al. [37]. The total reaction volume for the gastric phase was 30 mL, which consisted of 15 mL of the oil body suspension mixed with an equal volume of simulated gastric electrolyte solution. The pH of the mixture was adjusted to 2.0 using 1.0 mol/L HCl, followed by the addition of pepsin (2000 U/mg, based on protein content). The mixture was incubated in a shaker at 37 °C for 2 h. Subsequently, 15 mL of the simulated gastric digestion fluid was collected and heated in a boiling water bath for 5 min to terminate the digestion. After cooling, the mixture was centrifuged at 10,000× *g* for 10 min and stored at −20 °C. The remaining simulated gastric digestion fluid was mixed with an equal volume of simulated intestinal electrolyte solution. The pH was adjusted to 7.0 with 1.0 mol/L NaOH, after that trypsin (250 U/mg, based on protein content) was added. The mixture was further incubated at 37 °C for 2 h, then processed identically (boiling water bath, centrifugation, storage at −20 °C) to stop digestion.

### 4.4. Particle Size and ζ-Potential

A laser diffraction particle size analyzer (SYNC, Micro-Nano Particle Instrument Co., Ltd., Jinan, China) and ζ-potential analyzer (Malvern Instruments, Worcestershire, UK) were used to measure the particle size and ζ-potential in the oil body suspension. Before the analysis, the suspension was diluted 100-fold and 500-fold with a suitable phosphate-buffered solution for particle size and ζ-potential measurements, respectively. Control group refers to the original oil body suspensions without any digestion treatment, which were only diluted with phosphate-buffered solution.

### 4.5. Protein Hydrolysis Degree

The determination of protein hydrolysis degree was conducted according to the method described by Zhang et al. [38]. For the determination of protein content before digestion, samples were pre-frozen at −80 °C for 48 h and subsequently lyophilized in a vacuum freeze dryer to obtain lyophilized powders; these lyophilized samples were then dispersed in 4% (*w*/*v*) SDS solution, vortexed for 1 min, and centrifuged at 12,000 rpm and 4 °C for 40 min. After digestion, samples were diluted with 4% (*w*/*v*) SDS solution at a 2:1 ratio, vortexed for 1 min, and centrifuged at 12,000 rpm and 4 °C for 10 min. The supernatant was collected, and protein content was quantified using a commercial BCA protein assay kit. The degree of protein hydrolysis was calculated according to the formula provided below.

(1) $$Protein\;hydrolysis\;degree(\%)=\frac{x-x_{0}}{x}\times 100$$

where x is the protein content of the suspension before digestion, and x_0_ is the protein content of the suspension after digestion.

### 4.6. Determination of Free Amino Groups

The content of free amino groups was determined via the o-phthaldialdehyde (OPA) method, with modifications based on the protocol described by Liu et al. [39]. Briefly, 200 μL of the diluted oil body suspension was thoroughly mixed with 4 mL of OPA reagent, followed by incubation in a 35 °C water bath for 2 min. The absorbance of the resulting solution was measured at 340 nm. A standard curve was constructed with L-leucine (y = 11.535x + 0.1502, R^2^ = 0.9994), and the content of free amino groups was calculated according to this standard curve.

### 4.7. Release Rate of Free Fatty Acids

A pH meter was used to monitor the intestinal-phase lipid hydrolysis according to the method of Ye et al. [40]. Mixing the gastric phase with simulated intestinal fluid (SIF) until achieved the final ratio of 50:50 (*v*/*v*). Bile salts and CaCl_2_ were added until each reached 10 mM and 0.3 mM in the final mixture. Due to the low lipase activity (10 U mg^−1^) in the pancreatic enzyme extraction, the lipase activity was adjusted to 2000 U mL^−1^ by mixing pancreatic enzyme extract from porcine pancreas (4 × USP, P1750) with pure lipase from porcine pancreas Type II (100–500 U mg^−1^ protein) at a ratio of 10:1. The mixture was titrated with 1 mol/L NaOH every 20 min over 120 min, with the endpoint pH at 7.0. The formula is as follows:

(2) $$FFA(\%)=\frac{V_{NaOH}\times M_{NaOH}\times M_{Lipid}}{2\times W_{Lipid}}$$

where V_NaOH_ represents the volume of NaOH at pH 7.0, M_NaOH_ denotes the molar concentration of NaOH, M_Lipid_ stands for the average molecular weight of IPM oil, and W_Lipid_ indicates the total mass of IPM oil.

### 4.8. DPPH Radical Scavenging Activity

The DPPH radical scavenging activity was determined with a 96-well plate following the method described by Barreira et al. [41]. A DPPH working solution was prepared by dissolving DPPH reagent in methanol to a final concentration of 0.1 mM. Aliquots of the sample solution (100 μL) were mixed with 100 μL of the DPPH working solution in each well, followed by incubation at ambient temperature in the dark for 30 min. The reduction in DPPH radicals was measured at 517 nm using a microplate reader. A buffer blank was prepared without the addition of the sample. The DPPH radical scavenging activity was calculated using the following equation:

(3) $$DPPH\;radical\;scavenging\;activity=(1-\frac{A_{i}-A_{j}}{A_{0}})\times 100$$

where A_i_ is the absorbance value of the sample reacted with the DPPH solution, A_j_ is the absorbance value of the sample reacted with methanol, and A_0_ is the absorbance value of deionized water reacted with the DPPH solution.

### 4.9. Total Reducing Capacity

The total reducing power was determined according to the method described by Amarowicz et al. [42]. A 2.5 mL aliquot of each sample was mixed sequentially with 2.5 mL of 0.2 mol/L phosphate buffer (pH 6.6) and 2.5 mL of 1% potassium ferrocyanide solution. The mixture was incubated in a 50 °C water bath for 20 min and then rapidly cooled. Subsequently, 2.5 mL of 10% trichloroacetic acid solution was added, and the mixture was centrifuged at 3000 rpm for 10 min. A 5 mL portion of the supernatant was collected, followed by the addition of 4 mL of distilled water and 1 mL of 0.1% ferric chloride solution. After thorough mixing and a 10 min reaction, the absorbance of the mixture was measured at 700 nm. A higher absorbance value indicates a stronger reducing power.

### 4.10. α-Amylase Inhibitory Ability Analysis

The method reported by Cao et al. [12] was employed. A 0.2 mL of α-amylase solution (1 U/mL) reacted with 0.2 mL of sample solution in a 10 mL centrifuge tube for 10 min at 37 °C. Subsequently, oscillatory enzymatic hydrolysis was performed at 37 °C for 10 min after 0.3 mL of 1% soluble starch solution was added to the mixture. After that, 0.2 mL of DNS solution was added, and the mixture was boiled for 10 min before being adjusted to a volume of 4 mL. The absorbance value was measured at a wavelength of 540 nm. The solution without sample addition worked as the blank control group. The formula for calculating the α-amylase inhibition rate is as follows:

(4) $$\alpha -amylase\;inhibition\;rate=1-\frac{A_{3}-A_{4}}{A_{1}-A_{2}}\times 100$$

where A_1_ refers to the blank group, consisting of 0.2 mL α-amylase solution, 0.2 mL PBS, 0.3 mL starch solution, and 0.2 mL DNS reagent. A_2_ is the blank control group, which contains 0.4 mL PBS, 0.3 mL starch solution, and 0.2 mL DNS reagent. A_3_ represents the sample group, composed of 0.2 mL α-amylase solution, 0.2 mL sample, 0.3 mL starch solution, and 0.2 mL DNS reagent. A_4_ is the background control group, including 0.2 mL PBS, 0.2 mL sample, 0.3 mL starch solution, and 0.2 mL DNS reagent.

### 4.11. α-Glucosidase Inhibitory Ability Analysis

The α-glucosidase inhibitory activity assay followed the method described by Hu et al. [43]. A 1.0 mL of α-glucosidase solution (0.1 mol/L phosphate-buffered solution, pH 6.8, preserved at −20 °C) reacted with 0.5 mL sample solution for 10 min at 37 °C. Subsequently, 1.0 mL of 25 mmol/L PNPG (0.1 mol/L PBS, pH 6.8) was added to the mixture to conduct reaction for another 10 min at 37 °C. Finally, 1.0 mL of 0.1 mol/L anhydrous sodium carbonate was added to terminate the reaction, and the absorbance was measured at 405 nm. The solution without sample addition worked as the blank control group. Each experiment was conducted three times in parallel, and the average value was calculated. The α-glucosidase inhibition rate was calculated using the following formula:

(5) $$\alpha -glucosidase\;inhibition\;rate=1-\frac{A_{1}-A_{2}}{A_{3}-A_{4}}\times 100$$

where A_1_ corresponds to the sample solution; A_2_ refers to the system where α-glucosidase solution was replaced with buffer solution; A_3_ denotes the blank group, with the sample solution substituted by buffer solution; and A_4_ represents the control group, in which both the sample solution and α-glucosidase solution were replaced with buffer solution.

### 4.12. Statistical Analysis

All experiments were performed in at least three independent replicates. The experimental results were presented as average value ± standard deviation, and statistical analysis of the data was conducted using SPSS 25 software. Data were analyzed using a single-factor analysis of variance (ANOVA), and Duncan’s multiple range test was performed to compare the differences among variables. A value of *p* < 0.05 was considered statistically significant. All figures were plotted using Origin 2021.

## Data Availability

The original contributions presented in this study are included in the article. Further inquiries can be directed to the corresponding authors.

## Abbreviations

The following abbreviations are used in this manuscript:

IPM	*Idesia polycarpa* Maxim
EOB	Entire fruits oil body
POB	Peel oil body
SOB	Seed oil body
MGD	model of gastric digestion
MID	model of intestinal digestion

## References

[1] Hou K., Yang X., Bao M., Chen F., Tian H., Yang L. (2018). Composition, characteristics and antioxidant activities of fruit oils from *Idesia polycarpa* using homogenate-circulating ultrasound-assisted aqueous enzymatic extraction. Ind. Crop. Prod..

[2] Xiang X., Wen L., Wang Z., Yang G., Mao J., An X., Kan J. (2023). A comprehensive study on physicochemical properties, bioactive compounds, and emulsified lipid digestion characteristics of *Idesia polycarpa* var. *Vestita* Diels fruits oil. Food Chem..

[3] Zuo Y., Liu H., Li B., Zhao H., Li X., Chen J., Wang L., Zheng Q., He Y., Zhang J. (2024). The *Idesia polycarpa* genome provides insights into its evolution and oil biosynthesis. Cell Rep..

[4] Zhang W., Zhao C., Karrar E., Du M., Jin Q., Wang X. (2023). Analysis of Chemical Composition and Antioxidant Activity of *Idesia polycarpa* Pulp Oil from Five Regions in China. Foods.

[5] Huang L., Zeng Y., Li F., Zheng X., Rao Q., Gajendran B., Varier K.M., Peng T., Tang L. (2023). Polyphenolic compounds from *Idesia polycarpa* Maxim. fruits ameliorate non-alcoholic fatty liver disease by modulating lipid metabolism in oleic acid-induced HepG2 cells and high-fat diet-induced mice. J. Funct. Foods.

[6] An P., Wan S., Luo Y., Luo J., Zhang X., Zhou S., Xu T., He J., Mechanick J.I., Wu W.-C. (2022). Micronutrient Supplementation to Reduce Cardiovascular Risk. J. Am. Coll. Cardiol..

[7] Younes S. (2024). The role of micronutrients on the treatment of diabetes. Hum. Nutr. Metab..

[8] Shafras M., Sabaragamuwa R., Suwair M. (2024). Role of dietary antioxidants in diabetes: An overview. Food Chem. Adv..

[9] Ruban M., Pozhidaeva E., Bolotina L., Kaprin A. (2025). The Role of Diet and Nutrition in Cancer Development and Management: From Molecular Mechanisms to Personalized Interventions. Foods.

[10] German J.B., Dillard C.J. (2006). Composition, Structure and Absorption of Milk Lipids: A Source of Energy, Fat-Soluble Nutrients and Bioactive Molecules. Crit. Rev. Food Sci. Nutr..

[11] Abdullah, Weiss J., Zhang H. (2020). Recent advances in the composition, extraction and food applications of plant-derived oleosomes. Trends Food Sci. Technol..

[12] Cao J., Zhang J., Cao R., Zhang B., Miao M., Liu X., Sun L. (2024). Enzymolysis Modes Trigger Diversity in Inhibitor-α-Amylase Aggregating Behaviors and Activity Inhibition: A New Insight into Enzyme Inhibition. Adv. Sci..

[13] Liu Y., Zhang J., Liu X., Zhang J., Zhu W., Jiang Z., Feng H., Song M., Hou J., Chang Y. (2025). Physicochemical properties and stability of oil body in different parts of *Idesia polycarpa* Maxim. Sci. Technol. Food Ind..

[14] Zhang S., Chen H., Geng F., Peng D., Xie B., Sun Z., Chen Y., Deng Q. (2022). Natural oil bodies from typical oilseeds: Structural characterization and their potentials as natural delivery system for curcumin. Food Hydrocoll..

[15] Yang X., Wu Y., Liu Y., Ding X., Zhang D., Zhao L. (2022). Digestive characteristics of oil body extracted from soybean aqueous extract at different pHs. Food Res. Int..

[16] Liu B., Wu Y., Fang Y., Chen L., Ding X., Wang W., Zhao L. (2024). Physicochemical and digestive characteristics of high internal phase emulsion based on pumpkin seed oil bodies prepared at different pHs. LWT.

[17] Farooq S., Ahmad M.I., Ali U., Zhang H. (2024). Fabrication of curcumin-loaded oleogels using camellia oil bodies and gum arabic/chitosan coatings for controlled release applications. Int. J. Biol. Macromol..

[18] Rahman M., Farooq S. (2025). Role of peanut oleosomes in the delivery of curcumin embedded in interpenetrating emulsion-filled gels made with whey protein and chitosan. Colloids Surf. A Physicochem. Eng. Asp..

[19] Lee S., Jo K., Jeong S.-K.-C., Choi Y.-S., Jung S. (2023). Strategies for modulating the lipid digestion of emulsions in the gastrointestinal tract. Crit. Rev. Food Sci Nutr..

[20] Li T., Chen Y., Liu K. (2023). Research progress on the effect of interfacial proteins on the stability of emulsion during aqueous enzymatic oil extraction. Food Sci..

[21] Kou X., Hong M., Pan F., Huang X., Meng Q., Zhang Y., Ke Q. (2024). Inhibitory effects of nobiletin-mediated interfacial instability of bile salt emulsified oil droplets on lipid digestion. Food Chem..

[22] Li Y., Bao Z., Gu S., Wang Z., Zeng M., He Z., Chen Q., Qin F., Chen J. (2025). Effect of molecular weight and protein content on the interfacial activity of soybean soluble polysaccharides. Food Hydrocoll..

[23] Okuro P.K., Gomes A., Cunha R.L. (2023). Modulating digestion by composite interfacial layer in structured oil-in-water emulsions. Colloids Surf. A Physicochem. Eng. Asp..

[24] Chen L., Yokoyama W., Liang R., Tam C., Miller J., Zhong F. (2020). Remodeling of β-Carotene-Encapsulated Protein-Stabilized Nanoparticles during Gastrointestinal Digestion In Vitro and in Mice. J. Agric. Food Chem..

[25] Fan Y., Kou Z., Cao J., Wang Z., Zhang T., Han R., Che D. (2025). Dynamic Changes in Amino Acid Release Patterns of Different Plant Protein Sources During In Vitro Digestion and Their Nutritional Value Assessment. Animals.

[26] Ye Z., Cao C., Liu Y., Cao P., Li Q. (2018). Triglyceride Structure Modulates Gastrointestinal Digestion Fates of Lipids: A Comparative Study between Typical Edible Oils and Triglycerides Using Fully Designed In Vitro Digestion Model. J. Agric. Food Chem..

[27] Tian Y., Zhao X., Wang Z., Zhang W., Jiang Z. (2024). Structural characteristics and stability analysis of coconut oil body and its application for loading β-carotene. Food Chem..

[28] Chen Y., Lin Q., Wang J., Mu J., Liang Y. (2023). Proteins, polysaccharides and their derivatives as macromolecular antioxidant supplements: A review of in vitro screening methods and strategies. Int. J. Biol. Macromol..

[29] Zhu L., Chen J., Tang X., Xiong Y.L. (2008). Reducing, Radical Scavenging, and Chelation Properties of in Vitro Digests of Alcalase-Treated Zein Hydrolysates. J. Agric. Food Chem..

[30] You L., Zhao M., Regenstein J.M., Ren J. (2010). Changes in the antioxidant activity of loach (*Misgurnus anguillicaudatus*) protein hydrolysates during a simulated gastrointestinal digestion. Food Chem..

[31] Cheong A.M., Tan C.P., Nyam K.L. (2016). In-vitro gastrointestinal digestion of kenaf seed oil-in-water nanoemulsions. Ind. Crop. Prod..

[32] Valgimigli L., Banks J.T., Ingold K.U., Lusztyk J. (1995). Kinetic Solvent Effects on Hydroxylic Hydrogen Atom Abstractions Are Independent of the Nature of the Abstracting Radical. Two Extreme Tests Using Vitamin E and Phenol. J. Am. Chem. Soc..

[33] Lei J., Zhang H., Yan Q., Jiang Z., Chang C. (2025). Improving α-amylase inhibitory activity of simulated gastrointestinal digested pea protein by pH shifting assisted proteolysis. Food Chem..

[34] Yan X., Fan F., Qin Z., Zhang L., Guan S., Han S., Dong X., Chen H., Xu Z., Li T. (2024). Preparation and Characterization of Calcium-Chelated Sea Cucumber Ovum Hydrolysate and the Inhibitory Effect on α-Amylase. Foods.

[35] Zheng Y., Liu S., Xie J., Chen Y., Dong R., Zhang X., Liu S., Xie J., Hu X., Yu Q. (2020). Antioxidant, α-amylase and α-glucosidase inhibitory activities of bound polyphenols extracted from mung bean skin dietary fiber. LWT.

[36] Qian S., Wang G., Feng H., Chang Y., Lu J., Wang W., Hou J. (2025). Low-fat-high-stability non-dairy whipping cream prepared from soybean oil bodies as a substitute for hydrogenated oil: Impact of substitution ratio on structural properties, foam properties and sensory evaluation. Food Chem. X.

[37] Brodkorb A., Egger L., Alminger M., Alvito P., Assunção R., Ballance S., Bohn T., Bourlieu-Lacanal C., Boutrou R., Carrière F. (2019). INFOGEST static in vitro simulation of gastrointestinal food digestion. Nat. Protoc..

[38] Zhang R., Mu Z., Xu H., Yang N., Bilawal A., Jiang Z., Hou J. (2024). Insight into Sequential Extrusion and Cysteine Treatment for Improving Rheological Characteristics and In Vitro Digestibility of Whey Protein Isolate. J. Agric. Food Chem..

[39] Liu J., Song G., Yuan Y., Zhou L., Wang D., Yuan T., Li L., He G., Yang Q., Xiao G. (2022). Ultrasound-assisted assembly of β-lactoglobulin and chlorogenic acid for non covalent nanocomplex: Fabrication, characterization and potential biological function. Ultrason. Sonochem..

[40] Ye A., Cui J., Singh H. (2010). Effect of the fat globule membrane on in vitro digestion of milk fat globules with pancreatic lipase. Int. Dairy J..

[41] Barreira J., Ferreira I., Oliveira M., Pereira J. (2008). Antioxidant activities of the extracts from chestnut flower, leaf, skins and fruit. Food Chem..

[42] Amarowicz R., Pegg R.B., Rahimi-Moghaddam P., Barl B., Weil J.A. (2004). Free-radical scavenging capacity and antioxidant activity of selected plant species from the Canadian prairies. Food Chem..

[43] Hu H., Wang Y., Lu X. (2024). In vitro gastrointestinal digestion and colonic fermentation of media-milled black rice particle-stabilized Pickering emulsion: Phenolic release, bioactivity and prebiotic potential. Food Chem..

